# Measurement and control of bias in patient reported outcomes using multidimensional item response theory

**DOI:** 10.1186/s12874-016-0161-z

**Published:** 2016-05-26

**Authors:** N. Maritza Dowling, Daniel M. Bolt, Sien Deng, Chenxi Li

**Affiliations:** Department of Biostatistics and Medical Informatics, University of Wisconsin, Madison, WI USA; Wisconsin Alzheimer’s Disease Research Center, University of Wisconsin, Madison, WI USA; Department of Educational Psychology, University of Wisconsin, Madison, WI USA; Department of Epidemiology and Biostatistics, Michigan State University, East Lansing, MI USA

**Keywords:** Patient-reported outcomes (PROs), Extreme response style, Measurement invariance, Test validity, Multidimensional item response theory models

## Abstract

**Background:**

Patient-reported outcome (PRO) measures play a key role in the advancement of patient-centered care research. The accuracy of inferences, relevance of predictions, and the true nature of the associations made with PRO data depend on the validity of these measures. Errors inherent to self-report measures can seriously bias the estimation of constructs assessed by the scale. A well-documented disadvantage of self-report measures is their sensitivity to response style (RS) effects such as the respondent’s tendency to select the extremes of a rating scale. Although the biasing effect of extreme responding on constructs measured by self-reported tools has been widely acknowledged and studied across disciplines, little attention has been given to the development and systematic application of methodologies to assess and control for this effect in PRO measures.

**Methods:**

We review the methodological approaches that have been proposed to study extreme RS effects (ERS). We applied a multidimensional item response theory model to simultaneously estimate and correct for the impact of ERS on trait estimation in a PRO instrument. Model estimates were used to study the biasing effects of ERS on sum scores for individuals with the same amount of the targeted trait but different levels of ERS. We evaluated the effect of joint estimation of multiple scales and ERS on trait estimates and demonstrated the biasing effects of ERS on these trait estimates when used as explanatory variables.

**Results:**

A four-dimensional model accounting for ERS bias provided a better fit to the response data. Increasing levels of ERS showed bias in total scores as a function of trait estimates. The effect of ERS was greater when the pattern of extreme responding was the same across multiple scales modeled jointly. The estimated item category intercepts provided evidence of content independent category selection. Uncorrected trait estimates used as explanatory variables in prediction models showed downward bias.

**Conclusions:**

A comprehensive evaluation of the psychometric quality and soundness of PRO assessment measures should incorporate the study of ERS as a potential nuisance dimension affecting the accuracy and validity of scores and the impact of PRO data in clinical research and decision making.

## Background

Patient-reported outcomes (PROs) research relies on and is informed by the values, attitudes, and perceptions of patients throughout the research process. PROs are increasingly used in clinical trials as primary or key secondary outcomes to measure a wide range of health-related quality of life constructs and their determinants including the patients’ perspective of symptoms and the beneficial effects of drug therapies [1–3]. Data collected on these self-reported measures provide valuable input for assessing health status, informing clinical decision-making, and judging clinical improvement. The impact and recognized benefits of PROs in research, clinical practice, and patient-centered care quality has prompted several working groups to delineate guidelines and standards for the selection, design, and analysis of effective assessment measures (see e.g., [4–7]). Among the recommended “best practice” standards for research quality is the use of modern psychometric methods for scale development and analysis to enhance the precision, responsiveness, and validity of PROs measures.

In most PRO questionnaires, respondents are asked to rate their degree of agreement with a series of statements using a multipoint or Likert-type scaling format ranging, for example, from “strongly agree” to “strongly disagree.” A well-known disadvantage of self-rate or self-report measures, however, is their sensitivity to response style (RS) effects [8–10]. That is, other content-irrelevant or nuisance factors, such as personality traits, may systematically influence and distort responses to survey questions. This type of measurement bias can seriously affect the estimation of the targeted construct, and hence the validity of scale scores, and the application of psychometric models that assume invariance of item parameters across respondents and also assessment periods. Moreover, empirical evidence suggests that extreme response “tendencies” or styles are relatively stable and consistent both over different scales and across time [11–13].

Among the different types of RS behaviors, one of the most commonly discussed in the literature, and addressed in this study, is extreme response style (ERS). Comprehensive reviews of the different types of RS effects are provided by [8] and most recently by [14]. ERS reflects the tendency to select the extreme endpoints of a Likert-type or rating scale (e.g., 1s and 7s on a 7-point scale) regardless of the latent trait level or the specific item content [10, 15]. Ignoring ERS may affect summed scores causing a reordering of respondents at both ends of a scale and making low scores lower and high scores higher [8, 16, 17].

Several variables have been shown to be consistently related to ERS. For example, differences in extreme responding behavior have been associated with psychological traits such as anxiety [18, 19] and intelligence [20], demographic variables such as age and gender [15], and ethnic, socio-economic, and situational or cultural background (see e.g., [21–25]). The link between variability across the groups defined by these variables and RS tendencies in self-reporting may give rise to differential item function (DIF; [26]); a source of measurement bias well studied and identified in a wide range of PRO assessment tools [27–30]. It is possible that items in a self-report instrument identified as showing DIF relative to group membership reflect only group differences in RS behavior rather than the content or features of the item itself, which is the standard definition of DIF. Similarly, individuals located at the same trait level may receive different summed scores due primarily to RS differences not necessarily associated with membership in a manifest group. Bolt and Johnson [16] argue that to make accurate decisions on item modification and bias interpretation and elucidate the underlying causes of DIF, it is important to distinguish between two potential sources of DIF: (a) characteristics of the items that are related differentially to subgroups of respondents and (b) individual differences in the use of Likert-type scales (i.e., response style behavior). In fact, a recent study conducted by [31] found that gender-DIF and RS had an independent influence on item responses. Additionally, when authors controlled for ERS, the magnitude of DIF and the classification of items as DIF changed suggesting the importance of controlling for this confounder.

Although the biasing effect of ERS on constructs measured by self-reported assessments has been acknowledged and widely studied across disciplines since the late 40s (e.g., [32, 33]), little attention has been given to the development and/or systematic application of methodologies to specifically detect and control for this effect in PRO measures (see e.g., [34–37]). In this study, we present a general overview of the most common methods referenced in the literature and investigate the potential effects of ERS on trait estimates applying a multidimensional methodology to item-level rating scores from a widely-used PRO assessment tool in mental health: the NEO Five-Factor Inventory (NEO-FFI; [38]).

### Accounting for ERS

A number of approaches have been proposed in the literature for identifying, measuring, and controlling for ERS. To some extent, differences between the recommended methodology stem from different conceptualizations of ERS ranging from correcting and reducing its effect on the trait measured by the instrument to highlighting it by modeling its association with other variables of interest [39]. The most simple class of methods for operationalizing ERS have included summing up the unweighted or weighted frequencies of end-point responses into a single score or calculating the standard deviation from the mean scale score or from the mid-point of the scale [8]. An important drawback of these basic approaches is that it is difficult to disentangle the ERS index from the latent trait assessed by the scale. One proposed strategy to circumvent this limitation has been to include a set of items in the assessment instrument, designed specifically to measure ERS, to ensure that the content of the item is minimally confounded with extreme responding behavior [8, 9]. However, the selection and validation of items with the necessary characteristics to measure ERS (e.g., content heterogeneity, comparable pattern of frequencies, low inter-item correlations with the trait measured by the scale) may be a relatively cumbersome and impractical task in scale development [40].

A second class of approaches have used latent variable models such as mixture models to study latent groups of individuals representing different RS behaviors allowing a unidimensional trait estimation conditioned upon class membership (see e.g., [41–43]) and also the study of DIF within the identified latent groups [31]. This modeling approach assumes that ERS is a discrete or qualitative variable and individuals manifest one of several latent response styles. For example, Moors [41, 44] proposed a latent class factor analysis (LCFA) model that, unlike item response theory (IRT) models, treats latent variables as discrete (ordinal) and the rating scale items as nominal response variables. This approach allows the definition of latent variables for each substantive trait measured by the scale items and a separate factor measuring unobserved group heterogeneity in extreme response behavior and differential style effects across items. A multinomial logistic model is subsequently applied using individual item responses as outcomes and the estimated trait(s) and ERS latent classes as predictors. Variations of this modeling approach have employed a mixed polytomous Rasch model to a) test for the presence of latent classes of respondents displaying a differential use of the response scale and b) obtain parameter estimates within each class [31, 45]. Latent trait estimates, however, are assumed to be the same across classes and dissimilarities between latent classes are interpreted as the result of differences in RS behavior.

Although mixture modeling approaches are informative in revealing response pattern heterogeneity in the tested population and exploring differential style factor effects on scale items, they provide less information on how to correct the main trait estimates for the bias induced by the ERS factor. Other model-based approaches assume instead that a stable and latent continuous ERS trait underlies a person‘s response behavior, which is independent of the construct measured by the assessment tool [16, 46]. De Jong et al. [46], for example, proposed a multidimensional IRT (MIRT) model that yields separate Bayesian point estimates for latent continuous parameters measuring one dominant underlying trait and the RS effect. The model, though, requires the dichotomization of each scale item into “extreme” versus “remaining categories,” which may result in significant loss of information about the substantive trait being measured [47].

Alternative approaches within the MIRT framework jointly model multiple traits with differential influence on item response categories without the need to dichotomize the scale [16, 40, 48]. For instance, Bolt and Newton [40] introduced a flexible multidimensional nominal response model (MNRM), that allowed the simultaneous estimation of construct-related traits and extreme responding trait as separate dimensions accounting for the specific influence of these traits on response category selection. Information from each item category across scales is incorporated into the model. As was the case in Moor‘s (2003) approach, observed responses to the multi-category items are modeled using a multinomial logistic regression model. In this paper, we chose Bolt and Newton‘s approach to show and correct for the potential biasing effects of ERS on the primary trait estimates obtained from several self-reported measures of diverse content. The conceptual framework of many of the available PRO instruments is multidimensional with subscales assessing different aspects of health. The choice of the MNRM methodological approach seemed appropriate for this illustration.

It is important to note that recently introduced models have explicitly linked the study of response style to the theoretical underpinnings of the latent response process. In these models, responses to items are explained as a series of sequential decisions (see e.g., [17, 49]). For instance, the three-process model proposed by Bockenholt [49] uses IRT decision tree models to assess individual differences in the response processes underlying the person choice of specific options. While this methodology facilitates the decomposition of response processes into the targeted trait and individual response tendencies (e.g., ERS and acquiescence) providing insight into the independent effect of these processes on scale scores [50], it relies on strong assumptions on how the respondent moves through a decision-making process when answering Likert-type items.

## Methods

### The MNRM for detecting and correcting for ERS

Bolt and Newton [40] formulated the MNRM as:

1 $${} \begin{aligned}  &P(U_{j}=k\,|\,\theta_{l},\ldots,\theta_{m},\theta_{ERS})\\ &=\!\frac{exp(a_{jk1}\theta_{1}+\ldots+a_{jkm}\theta_{m} +a_{jk(m+1)}\theta_{ERS}+c_{jk})} {\sum_{h=1}^{k}exp(a_{jh1}\theta_{1}+\ldots+a_{jhm}\theta_{m} +a_{jh(m+1)}\theta_{ERS}+c_{jh})}, \end{aligned}  $$

for item *j*, category *k*, and *m+ERSθ* dimensions assumed to influence item responding and category selection. *θ*_1_,…, *θ*_*m*_ represent the substantive traits measured by the scale and *θ*_*ERS*_ denotes the ERS trait for all items in the scale. The probability *P* of selecting item category *k* is a function of *θ* dimensions, a discrimination or category slope parameter denoted as *a*, and an intercept parameter *c*. In the illustration that follows, we included three substantive traits and a response style trait. This MNRM allows the estimation of models with different constraints on the slope parameters. For example, if a block of items share the same rating scale, it can be assumed that the category slope parameters for a given trait is constant across those items. Alternatively, different category slope parameters can be specified for items within a latent trait dimension. This flexibility facilitates the estimation of correlations between the latent traits [48]. The adequacy of model fit using various constraints can be assessed through multiple model comparison information criteria.

For the models specified in this study, parameter estimates were obtained using a hybrid maximum likelihood estimation (MLE) that iteratively combined expectation maximization algorithms and modified Newton-Raphson methods. MLE utilized adaptive Gauss-Hermite numerical approximation with 10 quadrature nodes per model dimension. The procedure yields Bayesian expected a posteriori (EAP) estimates of the traits for each respondent. In secondary analyses presented as part of our illustration, EAP estimates were used as explanatory variables in a survival model. Further technical details on estimation are provided by Vermunt and Magidson [51]. All analyses were conducted using the Latent Gold 4.5 software [52].

### An empirical illustration

#### Background and data

We applied the MNRM approach to data from an ongoing clinicopathologic cohort study of incident Alzheimer’s disease (AD): the Religious Orders Study (ROS; [53]). ROS follow-up rate exceeds 95 % with up to 20 waves of data. Recruitment, exclusion, and inclusion criteria for this study and subject evaluations have been previously described in detail [54]. Briefly, ROS recruits older individuals without dementia who agree to receive clinical and psychological evaluation each year. Enrollment began in 1994 and includes the participation of over 1200 older religious clergy (priests, brothers, and nuns. The study was approved by the Institutional Review Board of Rush University Medical Center. Written informed consent was obtained from all study participants. The analysis included 1188 non-demented individuals who completed the NEO-FFI scale [38] as part of the assessment protocol at study entry. This sample represented approximately 97 % of the total study population. Participants were predominantly female (70 %), with a mean age at baseline of 73.81 (*SD*=6.71) and education level ranging from 3 to 18 years (*M*=18.42;*SD*=3.36).

We used the short version of the NEO-FFI consisting of 60 items mapping onto five 12-item dimensions representing personality constructs or traits (Neuroticism, Extraversion, Openness, Agreeableness, and Conscientiousness). All scale items were rated with a 5-point Likert scale (1 = *StronglyDisagree*, 2 = *Disagree*, 3 = *Neutral*, 4 =*Agree*, 5 = *StronglyAgree*). The observed responses may be viewed as a self-reported level of “symptom severity” with higher scores indicating more of the trait. For the purpose of this illustration, we studied ERS effects focusing on three dimensions (Neuroticism, Conscientiousness, and Agreeableness). Neuroticism measures susceptibility to psychological distress and tendency to negative affects. Conscientiousness items assess control of impulses, self-discipline, and determination. The Agreeableness dimension reflects altruistic behavior and eagerness to help others. The psychometric characteristics of the NEO scales have been extensively studied and found to be reliable and generalizable [55]. The scales have also been widely-used in population-based studies of mental disorders and associated with a range of clinical variables and comorbidities (see e.g., [56–58]).

## Results

### Model building and analysis results

To establish the presence of ERS as a dimension in the response data set, we specified a set of preliminary models with varying constraints. The first (baseline) MNRM included three dimensions: *θ*_*N*_, *θ*_*C*_, and *θ*_*A*_ corresponding to the main traits of interest, namely, ‘Neuroticism,’ ‘Conscientiousness,’ and ‘Agreeableness.’ In this model, the (response) category slope parameters, *a*_*jkm*_, as specified in Equation 1, were set to fixed equal interval values (−1,−0.5,0,0.5,1) across items for each construct. The formulation of this baseline model is similar to the multidimensional version of the partial credit model (PCM; [59]); one of the recommended models for evaluating PRO measures [60]. The second model added a fourth underlying dimension, potentially associated with ERS (*θ*_*ERS*_) bias. The category slopes specifying the fourth dimension were constrained to 0.75,−0.5,−0.5,−0.5, and 0.75 for all items in the scale. The equal positive values in the extreme categories and the negative values for the intermediate categories denote the slope parameters for the ERS trait.

Model selection and fit assessment were based on several penalized-likelihood information criteria, with lower values indicating a better fit. As explained in Vermunt and Magidson [51], the estimation of these indices is based on a log likelihood function (examining the likelihood of the data given the model parameters) and a penalty associated with model complexity. In Bayesian approaches to model selection, the log posterior probabilities of alternative models is used for the calculation of the information criterion. The log posterior is a function of the log likelihood and a prior probability distribution (log prior) selected to avoid boundary estimates. These information criteria included the Bayesian information criterion (BIC; [61]), the Akaike information criterion (AIC; [62]), and variations of the AIC index: the Akaike information criterion 3 (AIC3) and the consistent Akaike information criterion (CAIC).

All the fit indexes presented in Table 1 suggested that the four-dimensional model provided a better fit to the response data. A close examination of the fourth-factor category slopes across items showed a response pattern consistent with ERS. Therefore, the constraints imposed on the fourth factor representing the ERS trait seemed appropriate. As it is assumed in PCM models, the estimated category slopes for the first three targeted factors were roughly equally-spaced.

**Table 1:** Model comparison results

	Three-dimensional	Four-dimensional
	model	model with
		ERS constraints
# Par	148	149
Log-likelihood	–40525	–38518
Log-prior	–45	–48
Log-posterior	–40570	–38566
BIC	82107	78100
AIC	81346	77333
AIC3	81494	77482
CAIC	82255	78249

Note. BIC = Bayesian information criterion; AIC = Akaike information criterion; AIC3 = Akaike information criterion 3; CAIC = Consistent Akaike information criterion; ERS = Extreme response style

Table 2 summarizes the estimated category intercepts for the four-dimensional model. The category intercept provides information on the propensities towards item categories **unrelated** to the targeted trait. That is, intercepts reflect the likelihood of selecting an item category when the mean trait level is 0. Notice, for example, that the block of items measuring Conscientiousness (items 25–36) showed the highest positive intercept values on category 4 corresponding to ‘agree.’ With the exception of items 20 and 22, the same pattern is observed in the Agreeableness trait. Neuroticism appears to have a frequent content independent response of “2 = disagree.”

**Table 2:** Category intercept estimates for the four-dimensional model

		Category				
Trait	Item	1	2	3	4	5
	1	–0.971	**1.583**	0.385	1.153	–2.150
	2	–0.833	**2.262**	1.258	0.971	–3.659
	3	–1.281	**2.457**	0.978	1.224	–3.377
	4	–0.551	**2.607**	0.721	0.675	–3.452
	5	–0.571	**3.092**	1.318	0.762	–4.600
Neuroticism	6	0.685	**2.702**	0.507	0.524	–4.417
	7	–2.016	**2.366**	1.127	1.496	–2.972
	8	–0.251	**3.304**	1.193	0.208	–4.454
	9	–0.501	**3.390**	1.504	0.936	–5.329
	10	–1.103	**2.944**	1.023	1.044	–3.908
	11	0.686	**3.540**	1.151	0.611	–5.988
	12	0.044	**3.109**	0.753	0.715	–4.621
	13	–3.843	–1.539	–0.389	**3.514**	2.258
	14	–3.570	–0.478	0.490	**2.829**	0.730
	15	–4.542	0.997	1.349	**2.691**	–0.495
	16	–4.194	0.074	0.799	**3.059**	0.262
	17	–3.719	0.291	0.772	**2.556**	0.101
Agreeableness	18	–3.543	1.344	0.754	**2.465**	–1.019
	19	–4.470	–0.202	1.264	**3.847**	–0.439
	20	–1.189	0.118	**2.103**	-1.032	0.000
	21	–3.089	1.051	1.045	**2.270**	–1.277
	22	–3.822	–1.276	**3.632**	1.466	0.000
	23	–4.029	0.384	1.266	**2.982**	–0.604
	24	–5.305	0.181	0.832	**3.319**	0.973
	25	–3.710	0.265	0.776	**2.418**	0.252
	26	–3.815	0.122	0.505	**2.964**	0.223
	27	–3.561	0.686	0.800	**2.471**	–0.396
	28	–5.826	–1.564	–0.046	**4.588**	2.848
	29	–4.776	0.274	1.368	**3.085**	0.049
Conscientiousness	30	–4.359	0.869	0.916	**2.996**	–0.421
	31	–6.060	–0.827	0.893	**4.240**	1.755
	32	–5.423	–1.183	0.471	**4.133**	2.002
	33	–4.715	1.497	1.095	**2.918**	–0.795
	34	–6.140	0.072	1.711	**3.717**	0.639
	35	–4.710	0.606	0.894	**3.173**	0.038
	36	–5.167	0.626	1.692	**3.104**	–0.256

Note: Boldface numbers indicate the highest positive intercept values per item category

We used the MNRM estimates to study the biasing effects of extreme responding on sum scores for individuals with the same amount of the targeted trait but different levels of ERS. Using the Neuroticism scale for illustration, bias in a person’s sum score was calculated as a function of the person’s estimated levels on *θ*_*N*_ and *θ*_*ERS*_. The expected or "purified" sum score was expressed as

2 $$  ES(\theta_{N},\theta_{ERS})=\sum\limits_{j=1}^{12}\sum\limits_{k=1}^{5}k \times P(U_{j}=k\,|\,\theta_{N},\theta_{ERS}),  $$

with *P*(*U*_*j*_=*k* | *θ*_*N*_,*θ*_*ERS*_) defined by the MNRM parameter estimates obtained from Model 2. Assuming a mean of 0 for *θ*_*ERS*_ as a reference point and using the results from Eq. 2, bias was estimated as

3 $$  BIAS(\theta_{N},\theta_{ERS})=ES(\theta_{N},\theta_{ERS})-ES(\theta_{N},0).  $$

**Fig. 1:**
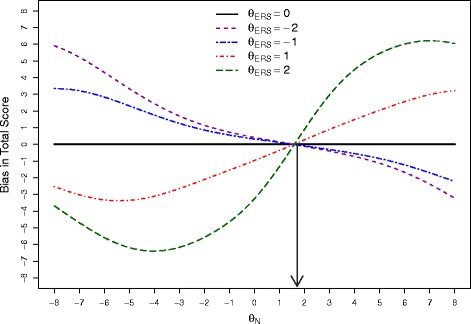
Bias in total scores as a function of *θ*_*N*_. The dotted line to the *θ*_*N*_ axis represent the point at which the mean expected score across all items is close to the midpoint in the 5-point Likert scale. For these data, this point is approximately 1.8

The magnitude of effects due to ERS can be assessed by inspecting the estimated bias relative to sum scores. Fig. 1 displays the bias in sum scores as a function of Neuroticism, *θ*_*N*_, at increasing levels of *θ*_*ERS*_=2,1,0,−1 and −2; with ‘2’ as the highest value.

Note that the bias is 0 for all levels of *θ*_*ERS*_ at the lines intersection point (roughly 1.8 on the *θ*_*N*_ scale). In this particular data set, 1.8 is also the estimated *θ*_*N*_ level at which the mean expected score across all items is close to 3; the midpoint of the 5-point Likert scale. With *θ*_*N*_ levels above 1.8, the expected item scores, on average, increase. This raises the likelihood of choosing upper end categories (4’s and 5’s) for those with extreme response tendencies. Conversely, individuals with lower levels of Neuroticism will be be located below 1.8 in the *θ*_*N*_ scale with average expected item scores also below category “3.” Consequently, respondents with extreme responding behavior will be more prone to select 1s and 2s.

It has been previously demonstrated that the joint estimation of multiple scales and ERS leads to higher accuracy in the identification of ERS and improved estimates of the targeted traits [16, 40]. The simultaneous analysis of multiple scales takes into account all the response patterns across scales in the estimation process. To illustrate the impact of joint modeling on parameter estimates, we examined differences in results when analyzing the three scales jointly with and without ERS included in the model. Table 3 compares trait estimates for a sample of persons with different patterns of “extremeness” across traits. For example, the scores on the Neuroticism scale (N) for first two cases may indicate ERS bias. The second pair (154 and 202) on the same trait shows a less extreme pattern. Regardless of the estimation model, and as expected, more extreme item categories tend to yield higher estimated $\hat {\theta }_{ERS}$. Results showed that the joint scale analysis yielded more variability in $\hat {\theta }_{N}$ estimates for respondents with similar $\hat {\theta }_{N}$ estimate in the 3-dimensional model. Note, for example, that case 885, with more extreme responses across all scales, obtained a lower (in absolute value) $\hat {\theta }_{N}$ estimate in the corrected model compared to case 151, with a less extreme pattern in the Agreeableness and Conscientiousness scales.

**Table 3:** Comparison of specific estimates for different response patterns across subscales

	Response vector			3-Dimensional model			4-Dimensional model			
Case#	N	A	C	$\hat {\theta }_{N}$	$\hat {\theta }_{A}$	$\hat {\theta }_{C}$	$\hat {\theta }_{N}$	$\hat {\theta }_{A}$	$\hat {\theta }_{C}$	$\hat {\theta }_{ERS}$
885	111111111111	555555555555	555553553555	**–7.10**	5.26	5.82	**–2.23**	1.47	0.30	3.71
151	111111111111	544555555555	442443452444	**–6.17**	3.81	-0.03	**–3.59**	1.80	–0.98	2.15
154	222222222222	444444444444	444444444444	**–1.09**	0.41	0.59	**–1.93**	0.92	1.65	–1.13
202	222222222222	444445445454	345443254332	**–1.06**	0.85	–1.38	**–0.99**	0.65	–1.14	0.88

Note: N = Neuroticism; A = Agreeableness; C = Conscientiousness; ERS = Extreme response style. Estimates for Neuroticism are indicated in boldface type

### Effect of ERS in prediction models

A number of studies have associated Neuroticism with cognitive decline, dementia, and increased risk of AD [63]. To evaluate the potential effects of controlling for ERS on risk prediction, we applied a Cox proportional hazards model [64] using the bias-corrected 4-dimensional MNRM (Model 2) parameter estimates for Neuroticism as predictors and time to AD conversion as the event of interest. The results were compared to estimates from a 3-dimensional MNRM (Model 1, not corrected for ERS). We also fitted a third Cox proportional hazards model using raw Neuroticism scores as predictors. All models controlled for gender, age, and years of education. To account for the uncertainty of the estimations obtained from the bias-corrected and bias-uncorrected MNRM models, we used 5 random draws from the posterior predictive distribution of the latent parameter estimates. The parameter estimates were then aggregated across the Cox regression analyses using the [65] formulae for summarizing multiple imputations that combines variability within and between data sets.

There were two possible events during follow-up in the data used in this illustration: conversion to AD and death. Therefore, we treated this dependent censoring as competing risks in the Cox regression models. This approach produces estimates of the cause-specific hazard of AD, which is not the same as the marginal hazard of time to AD [66]. Overall, the inspection of Cox-Snell residuals revealed that the models fit the data reasonably well.

As shown in Table 4, the models not adjusted for ERS underestimate the cause-specific hazard ratio. For example, in Model 1, for every unit increase in Neuroticism (*θ*_*N*_), the cause-specific hazard of progression to AD increased by 17.59 % (95 % confidence interval [CI], 1.082 – 1.285). In contrast, the model corrected for ERS effects, yielded an estimate of 21.53 % increase in risk of AD (95 % CI; 1.115 – 1.324). Although using a different metric, the model using ‘raw’ or sum scores on the Neuroticism scale as a predictor of the cause-specific hazard of AD conversion produced a relatively low hazard ratio estimate (4.1 %; 95 % CI; 1.015 – 1.069). The AIC fit index favored the corrected model (Model 2; AIC = 1973.08) over the uncorrected model (Model 1; AIC =1975.92) and the the model using raw Neuroticism scores as explanatory variable (AIC =1996.45). These results suggest that for this sample, the Neuroticism scale had the tendency, on average, to elicit ‘disagree’ responses across items. Therefore, ERS produced more extreme levels of disagreement with estimates of the substantive trait showing downward bias. The increase in estimation accuracy and efficiency produced by the multidimensional models, however, has an effect on the estimated standard errors (SEs) and corresponding 95 % CIs. Note that the estimated SE for Neuroticism is higher and the corresponding CI is wider in the model adjusted for ERS effects.

**Table 4:** Comparison of results from the Cox proportional hazards models with competing risk data

	Model using raw scores				Model not adjusted for ERS (Model 1)				Model adjusted for ERS (Model 2)			
Predictor	Hazard Ratio	SE	*p*-value	95 *%* CI	Hazard Ratio	SE	*p*-value	95 *%* CI	Hazard Ratio	SE	*p*-value	95 *%* CI
Age	**1.143**	0.012	<0.001	[1.116,1.169]	**1.141**	0.008	<0.001	[1.125,1.162]	**1.144**	0.012	<0.001	[1.117,1.171]
Male	1.059	0.177	0.746	[0.749,1.498]	0.961	0.134	0.694	[0.734,1.229]	1.069	0.177	0.707	[0.755,1.513]
Education	1.040	0.022	0.067	[0.997,1.088]	1.042	0.022	0.064	[0.998,1.089]	0.956	0.132	0.734	[0.739,1.237]
Neuroticism	**1.041**	0.013	0.003	[1.014,1.069]	**1.176**	0.044	<0.001	[1.082,1.285]	**1.215**	0.044	<0.001	[1.115,1.324]

Notes. SE = Standard error; CI = Confidence interval; ERS = Extreme response style. Values that are statistically significant are indicated in bold

**Fig. 2:**
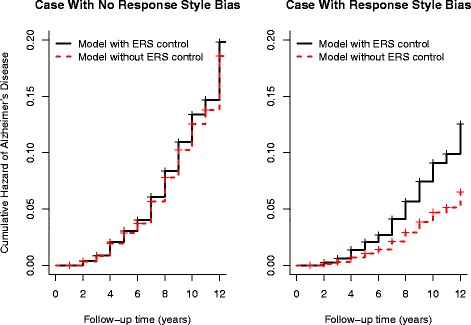
Cumulative hazard for the risk of incident Alzheimer’s disease (AD) for two cases in the sample. The first graph illustrates the cumulative hazard for an individual with a ‘non-extreme’ response pattern across scales using estimates of Neuroticism obtained from the multidimensional nominal response model (MNRM) adjusted (*solid lines*) and not adjusted (*dotted lines*) for extreme response style (ERS). The second graph shows the cumulative hazard for a second individual with an extreme response pattern using MNRM models controlling and not controlling for ERS effects

Figure 2 illustrates the estimates from the adjusted for ERS and non-adjusted models of the cumulative hazards of incident AD associated with a case exhibiting a “non-extreme” response pattern across constructs with a high level of Neuroticism and a second case showing an “extreme” response pattern across constructs with a low “trait” estimate on the Neuroticism scale. The two cases converted to AD during the course of the study and were matched on age, education, and gender. In this particular example, controlling for the biasing effects of ERS appears to have the greatest impact for an individual located in the lowest end of the *θ*_*N*_ distribution. That is, an individual responding in the lowest extreme categories of the scale (‘strongly disagree’ and ’disagree’).

## Discussion

The use of analytical approaches to minimize all forms of bias and increase validity is a key component of published guidelines for the development, evaluation, and score interpretation of PRO assessment instruments. The overall contribution of PRO data to patient-centered research greatly depends upon the psychometric quality of these measures. Although other forms of bias such as DIF has been extensively studied in the PRO literature, the investigation of extreme response behavior and the control for its effects have received less attention. To address this gap, this study provided an overview of a range of procedures to assess ERS tendencies in self-report measures and illustrated the application of a methodological approach to estimate and control for the potential biasing effects of ERS on substantive trait estimates. The application of model-based approaches to minimize invariance due to ERS is especially relevant in PRO measures, where unobserved personality factors (not targeted by the scale) are more likely to increase noise and reduce measurement precision affecting in turn the responsiveness or sensitivity of outcome measures. Meaningful and actionable self-reports of constructs across domains such as patient satisfaction with health care, level of pain, depression symptoms, and many other quality of life variables, are pivotal to patient-centered outcomes research.

Knowledge of statistical tools available to reduce the influence of confounders can only increase the accuracy and efficiency of PRO data and the inferential power of estimates obtained from models using these measures. Several recent simulation studies have demonstrated the potential of modern psychometric methods to study PROs in the field of clinical research (see e.g., [67, 68]). PRO instruments are inherently multidimensional and may comprise subscales assessing different aspects of health. When PRO scales consist of non-identical but correlated traits, the application of multidimensional IRT models has the benefit of facilitating the use of statistical information from all sets of items in the scale increasing the precision of latent scores, while controlling for irrelevant nuisance factors associated with response style. As argued by [16], extreme responding behavior may cause DIF across groups and should be accounted for when evaluating DIF hypotheses to minimize confounding.

We provided a detailed exposition of the MNRM approach for detecting and adjusting for ERS proposed by Bolt and Newton [40] using data from the NEO-FFI; a popular PRO measure in health care. We showed the gain in measurement accuracy of trait estimates when ERS was controlled for and the advantages of jointly estimating all the traits measured by the scale. Joint estimation utilizes information from all the subscales or dimensions measured by the instrument providing better control of ERS with respect to measurement of the substantive traits. The bias introduced by response style confounders can seriously affect individual trait estimates and distort their association with other outcomes of clinical importance. Using a real data set we demonstrated how ERS-induced bias may underestimate effect sizes and affect the association between PRO measures and the cause-specific hazard of AD conversion based on a Cox proportional hazards model. Effect sizes produced by simple sum scores of the targeted trait were relatively small compared to those produced by the MNRM for estimating trait parameters. These results suggest that accounting for ERS behavior using multidimensional IRT approaches may substantially increase the value of PRO measures as cogent evidence to support decision making in clinical and health policy development.

Efforts are currently underway to extend the MNRM approach presented in this study to allow the examination of ERS using longitudinal self-reported data. Detecting and controlling for longitudinal ERS bias can help improve the validity and sensitivity of PRO performance measures for the study of change across time and, hence, their value as adjunctive or primary measures in clinical trials. An increased interest in the use of PRO measures in cross-national or multi-country research has also heightened the need to develop valid international assessment tools to make meaningful comparisons between and within countries [69–71]. The development of a core set of standardized PRO measures ensuring conceptual equivalence across countries can be greatly enhanced by the use of methodologies in the calibration and validation process that allow the examination of ERS bias effects.

Cross-cultural variability in response styles has been extensively studied and well-documented [11, 24, 47, 72]. Country-specific variations in ERS may influence the interpretation of observed differences in the constructs measured by the instrument. Methodological approaches that integrate IRT measurement models and structural hierarchical models have been proposed to study the determinants of ERS across people and countries (see e.g., [46]). The estimated ERS scores can subsequently be used to adjust the data obtained from the assessment tool for ERS bias. Recently, Lu and Bolt [73] proposed a multilevel multidimensional IRT model that accommodates nested data (respondents within countries) and simultaneously detects and adjusts for ERS effects on the substantive trait estimates measured by the assessment instrument at both the respondent and country level.

There are other potential extensions of the MNRM model considered in this paper that could be applied with these data. Falk and Cai [74] proposed a model that includes an item-level discrimination parameter on the response style trait, allowing items to be differentially influenced by response styles. Besides being a more flexible model, a detailed study of response style discrimination could also inform the development of items that might minimize the influence of response style effects. For example, it might be anticipated that less ambiguous rating scale anchors, such as those that might attend to the frequencies of particular behaviors rather than specific levels of agreement, might result in more objective responses less subject to individual response tendencies.

Importantly, our paper focuses on just one form of response style that may contribute to bias in scale scores, namely extreme response style. Our interest in this form of response style is motivated by its frequent presence in scales of this kind, its known effects in contributing to bias, and its tendency to correlate with other person characteristics, which makes the bias of potentially greater consequence. Methods for attending to other forms of response style bias (see e.g., [74, 75]) exist and could also be considered. Moreover, other forms of bias unrelated to response style can naturally also be present. Methods for the exploratory study of differential item functioning (DIF) have become increasingly popular (see e.g., [76]), and may be helpful in this regard.

## Conclusion

Self-report is an integral component of the data-collection methodology in patient-centered research. This study has shown the importance of assessing and correcting for the idiosyncratic biases of self-reported measures that affect the validity, responsiveness, and impact of PRO instruments. It is therefore recommended that methods for ERS detection and control receive more attention in PRO assessment literature.

## Abbreviations

PRO, patient-reported outcome; RS, response style; ERS, extreme response style; DIF, diferential item function; NEO-FFI, NEO five factor inventory; MNRM, multidimensional nominal response model; MIRT, multidimensional item response theory; AD, alzheimer’s disease.

## References

[1] Basch E (2010). The missing voice of patients in drug safety reporting. N Engl J Med.

[2] Calvert M, Blazeby J, Altman DG, Revicki DA, Moher D, Brundage MD (2013). Reporting of patient-reported outcomes in randomized trials: the CONSORT PRO extension. N Engl J Med.

[3] Hobart JC, Cano SJ, Zajicek JP, Thompson AJ (1950). Rating scales as outcome measures for clinical trials in neurology: problems, solutions, and recommendations. Lancet Neurol.

[4] Basch E, Torda P, Adams K (2013). Standards for patient-reported outcome-based performance measures. J Am Med Assoc.

[5] Fries JF, Bruce B, Cella D (2005). The promise of PROMIS: using item response theory to improve assessment of patient-reported outcomes. Clin Exp Rheumatol.

[6] Reeve BB, Wyrwich KW, Wu AW, Velikova G, Terwee CB, Snyder CF (2013). ISOQOL recommends minimum standards for patient-reported outcome measures used in patient-centered outcomes and comparative effectiveness research. Qual Life Res.

[7] PROMIS Instrument Development and Psychometric Evaluation Scientific Standards. 2012. Available at: http://www.nihpromis.org/Documents/PROMISStandards_Vers2.0_Final.pdf.

[8] Baumgartner H, Steenkamp JB (2001). Response styles in marketing research: A cross-national investigation. J Marketing Res.

[9] Greenleaf EA (1992). Improving rating scale measures by detecting and correcting bias components in some response styles. J Market Res.

[10] Paulhus DL, Robinson JP, Shaver PR, Wrightsman LS (1991). Measurement and control of response bias. Measures of Personality and Social Attitudes.

[11] Clarke I (2001). Extreme response style in cross-cultural research. Int Market Rev.

[12] Kieruj ND, Moors G (2010). Variations in response style behavior by response scale format in attitude research. Int J Public Opin Res.

[13] Wetzel E, Lüdtke O, Zettler I, Bohnke JR (2015). The stability of extreme response style and acquiescence over 8 years. Assessment.

[14] van Vaerenbergh Y, Thomas TD (2013). Response styles in survey research: A literature review of antecedents, consequences, and remedies. Int J Public Opin Res.

[15] Greenleaf EA (1992). Measuring extreme response style. Public Opin Q.

[16] Bolt DM, Johnson TR (2009). Addressing score bias and differential item functioning due to individual differences in response style. Appl Psychol Meas.

[17] Thissen-Roe A, Thissen D (2013). A two-decision model for responses to likert-type items. J Educ Behav Stat.

[18] Hamilton DC (1968). Personality attributes associated with extreme response style. Psychol Bull.

[19] Plieger T, Montag C, Felten A, Reuter M (2014). The serotonin transporter polymorphism (5-httlpr) and personality: response style as a new endophenotype for anxiety. Int J Neuropsychopharmacol.

[20] Meisenberg G, Williams A (2008). Are acquiescent and extreme response styles related to low intelligence and education?. Pers Individ Diff.

[21] Azocar F, Areán P, Miranda J, Muñoz RF (2001). Differential item functioning in a spanish translation of the beck depression inventory. J Clin Psychol.

[22] Bachman J. G, O’Malley P. M. Response styles revisited: racial/ethnic and gender differences in extreme responding. 2010. Retrieved from http://monitoringthefuture.org/pubs/occpapers/occ72.pdf.

[23] Hamamura T, Heine SJ, Paulhus DL (2008). Cultural differences in response styles: The role of dialectical thinking. Pers Ind Diff.

[24] Harzing AW (2006). Response styles in cross-national survey research: A 26-country study. Int J Cross Cultural Manage.

[25] Marin G, Gamba RJ, Marin BV (1992). Extreme response style and acquiescence among hispanics:the role of acculturation and education. J Cross-Cultural Psychol.

[26] Holland PW, Wainer H (2012). Differential Item Functioning.

[27] McHorney CA, Fleishman JA (2006). Assessing and understanding measurement equivalence in health outcome measures. Medical Care.

[28] Teresi JA, Ramirez M, Lai JS, Silver S (2008). Occurrences and sources of differential item functioning (dif) in patient-reported outcome measures: Description of dif methods, and review of measures of depression, quality of life and general health. Psychol Sci Q.

[29] Teresi JA, Ocepek-Welikson K, Kleinman M (2009). Analysis of differential item functioning in the depression item bank from the patient reported outcome measurement information system (PROMIS): An item response theory approachn. Psychol Sci Q.

[30] Varni JW, Thissen D, Stucky BD (2014). PROMIS parent proxy report scales for children ages 5–7 years: An item response theory analysis of differential item functioning across age groups. J Cross-Cultural Psychol.

[31] Wetzel E, Böhnke J, Carstensen CH, Ziegler M, Ostendorf F (2013). Do individual response styles matter? assessing differential item functioning for men and women in the NEO-PI-R. J Ind Diff.

[32] Cronbach LJ (1946). Response sets and test validity. Educ Psychol Meas.

[33] Cronbach LJ (1950). Further evidence of response set test design. Educ Psychol Meas.

[34] Böhnke JR, Croudace TJ (2015). Factors of psychological distress: clinical value, measurement substance, and methodological artefacts. Soc Psychiatry Psychiatr Epidemiol.

[35] Elliott MN, Haviland AM, Kanouse D, Hambarsoomian K, Hays R (2009). Adjusting for subgroup differences in extreme response tendency in ratings of health care: impact on disparity estimates. Health Services Res.

[36] Peterson TJ, Feldman G, Harley R, Fresco DM, Graves L, Holmes A, Bogdan R, Papakostas G, Bohn L, Lury R (2007). Extreme response style in recurrent and chronically depressed patients: Change with antidepressant administration and stability during continuation treatment. J Consult Clinical Psychol.

[37] Weech-Maldonado R, Elliott MN, Oluwole A, Schiller K, Hays R (2008). Survey response style and differential use of CHAPS rating scales by hispanics. Med Care.

[38] Costa PT, McCrae RR (1992). NEO PI-R Professional Manual: Revised NEO Personality Inventory (NEO PI-R) and NEO Five-Factor Inventory (NEO-FFI).

[39] Moors G (2008). Exploring the effect of a middle response category on response style in attitude measurement. Qual Quantity.

[40] Bolt DM, Newton JR (2011). Multiscale measurement of extreme response style. Educ Psychol Meas.

[41] Moors G (2003). Diagnosing response style behavior by means of a latent-class factor approach: Sociodemographic correlates of gender role attitudes and perceptions of ethnic discrimination reexamined. Qual Quantity.

[42] Rost J, Carstensen CH, von Davier M, Rost J, Langeheine R (1997). Applying the mixed rasch model to personality questionnaires. Applications of Latent Trait and Latent Class Models in the Social Sciences.

[43] van Rosmalen J, van Herk H, Groenen PJF (2010). Identifying response styles: A latent-class bilinear multinomial logit model. J Market Res.

[44] Moors G (2004). Facts and artifacts in the comparison of attitudes among ethnic minorities. a multigroup latent class structure model with adjustment for response style behavior. Eur Sociol Rev.

[45] Rost J (1990). Rasch models in latent classes: An integration of two approaches to item analysis. Appl Psychol Meas.

[46] De Jong MG, Steenkamp J, Fox J, Baumgartner H (2008). Using item response theory to measure extreme response style in marketing research: A global investigation. J Market Res.

[47] Morren M, Gelissen J, Vermunt JK (2011). Dealing with extreme response style in cross-cultural research: A restricted latent class factor analysis approach. Sociol Methodol.

[48] Johnson TR, Bolt DM (2010). On the use of factor-analytic multinomial logit item response models to account for individual differences in response style. J Educ Behav Stat.

[49] Böckenholt U (2012). Modeling multiple response processes in judgment and choice. Psychol Methods.

[50] Zettler I, Lang J, Hülsheger UR, Hilbig BE. Dissociating indifferent, directional, and extreme responding in personality data: Applying the three-process model to self-and observer reports. J Pers. 2015. Advance online publication. doi:10.1111/jopy.12172. 10.1111/jopy.12172 25765765

[51] Vermunt JK, Magidson J (2013). Technical guide for Latent GOLD 5.0: Basic, advanced, and syntax.

[52] Vermunt JK, Magidson J (2013). Latent GOLD 5.0 upgrade manual.

[53] Bennett DA, Schneider JA, Arvanitakis Z, Wilson RS (2012). Overview and findings from the religious orders study. Curr Alzheimer Res.

[54] Wilson RS, Beckett LA, Barnes LL, Schneider JA, Bach J, Evans DA, Bennett DA (2002). Individual differences in rates of change in cognitive abilities of older persons. Psychol Aging.

[55] McCrae RR, Kurtz JE, Yamagata S, Terracciano A (2011). Internal consistency, retest reliability, and their implications for personality scale validity. Personal Soc Psychol Rev.

[56] Aldinger M, Stopsack M, Ulrich I, Appel K, Reinelt E, Wolff S, Grabe HJ, Lang S, Barnow S (2014). Neuroticism developmental courses-implications for depression, anxiety and everyday emotional experience; a prospective study from adolescence to young adulthood. BMC Psychiatry.

[57] Goodwin RD, Stein MB (2003). Peptic ulcer disease and neuroticism in the united states adult population. Psychother Psychosom.

[58] Kendler KS, Gatz M, Gardner CO, Pedersen NL (2006). Personality and major depression: a swedish longitudinal, population-based twin study. Arch Gen Psychiat.

[59] Muraki E (1992). Ia generalized partial credit model: Application of an em algorithm. ETS Res Report Ser.

[60] Li Y, Baser R (2012). Using R and WinBUGS to fit a generalized partial credit model for developing and evaluating patient-reported outcomes assessments. Stat Med.

[61] Schwarz G (1978). Estimating the dimension of a model. Ann Stat.

[62] Akaike H (1974). A new look at the statistical model identification. IEEE Trans Automatic Cont.

[63] Terracciano A, Sutin AR, An Y, O’Brien R, Zonderman AB, Resnick SM (2014). Personality and risk of alzheimer’s disease: New data and meta-analysis. Alzheimers Dement.

[64] Cox D (1984). Analysis of Survival Data.

[65] Little RJA, Rubin DB (2007). Statistical Analysis with Missing Data.

[66] Dignam JJ, Kocherginsky MN (2008). Choice and interpretation of statistical tests used when competing risks are present. J Clin Oncol.

[67] Blanchin M, Hardouin JB, Neel TL, Kubis G, Blanchard C, Mirallié E, Sébille V (2011). Comparison of ctt and rasch-based approaches for the analysis of longitudinal patient reported outcomes. Stat Med.

[68] Bock E, Hardouin JB, Blanchin M, Le Neel T, Kubis G, Bonnaud-Antignac A, Dantan E, Sébille V. Rasch-family models are more valuable than score based approaches for analysing longitudinal patient-reported outcomes with missing data. Statistical Methods in Medical Research. 2013. Advance online publication. doi:10.1177/0962280213515570. 10.1177/0962280213515570 24346165

[69] Alonso J, Bartlett SJ, Rose M, Aaronson NK, Chaplin JE, Efficace F, Leplège A, Lu A, Tulsky DS, Raat H, Ravens-Sieberer U, Revicki D, Terwee CB, Valderas JM, Cella D, Forrest CB (2013). The case for an international patient-reported outcomes measurement information system (promis®;) initiative. Health Qual Life Outcomes.

[70] Janssens A, Rogers M, Coon JT, Allen K, Green C, Jenkinson C, Tennant A, Logan S, Morris C (2015). A systematic review of generic multidimensional patient-reported outcome measures for children, part ii: evaluation of psychometric performance of english-language versions in a general population. Value Health.

[71] Watt T, Barbesino G, Bjorner JB, Bonnema SJ, Bukvic B, Drummond R, Groenvold M, Hegedüs L, Kantzer V, Lasch KE, Mishra A, Netea-Maier R, Ekker M, Paunovic I, Quinn TJ, Rasmussen K, Russell A, Sabaretnam M, Smit J, Torring O, Zivaljevic V, Feldt-Rasmussen U (2005). Cross-cultural validity of the thyroid-specific quality-of-life patient-reported outcome measure, thypro. Qual Life Res.

[72] Johnson T, Kulesa P, Cho Y, Shavitt S (2005). The relation between culture and response styles evidence from 19 countries. J Cross-cultural Psychol.

[73] Lu Y, Bolt DM (2015). Examining the attitude-achievement paradox in pisa using a multilevel multidimensional irt model for extreme response style. Large-scale Assessments Educ.

[74] Falk CF, Cai L. A flexible full-information approach to the modeling of response styles. Psychological Methods. 2015. Advance online publication. doi:10.1037/met0000059. 10.1037/met0000059 26641273

[75] Bolt DM, Lu Y, Kim JS (2014). Measurement and control of response styles using anchoring vignettes: A model-based approach. Psychol Methods.

[76] Strobl C, Kopf J, Zeileis A (2015). Rasch trees: A new method for detecting differential item functioning in the rasch model. Psychometrika.

